# Standardized Electric-Field-Resolved Molecular Fingerprinting

**DOI:** 10.1021/acs.analchem.4c01745

**Published:** 2024-07-29

**Authors:** Marinus Huber, M. Trubetskov, W. Schweinberger, P. Jacob, M. Zigman, F. Krausz, I. Pupeza

**Affiliations:** † Max Planck Institute of Quantum Optics, 85748 Garching, Germany; ‡ Department of Physics, Ludwig Maximilian University of Munich, 85748 Garching, Germany; § Leibniz Institute of Photonic Technology—Member of the Research Alliance, Leibniz Health Technologies, 07745 Jena, Germany; ∥ Cluster of Excellence Balance of the Microverse, Friedrich Schiller University Jena, 07743 Jena, Germany; ⊥ Physics Department and State Research Center OPTIMAS, University of Kaiserslautern-Landau, 67663 Kaiserslautern, Germany; # Center for Molecular Fingerprinting, 1093 Budapest, Hungary; ∇ Fraunhofer Institute for Industrial Mathematics ITWM, 67663 Kaiserslautern, Germany

## Abstract

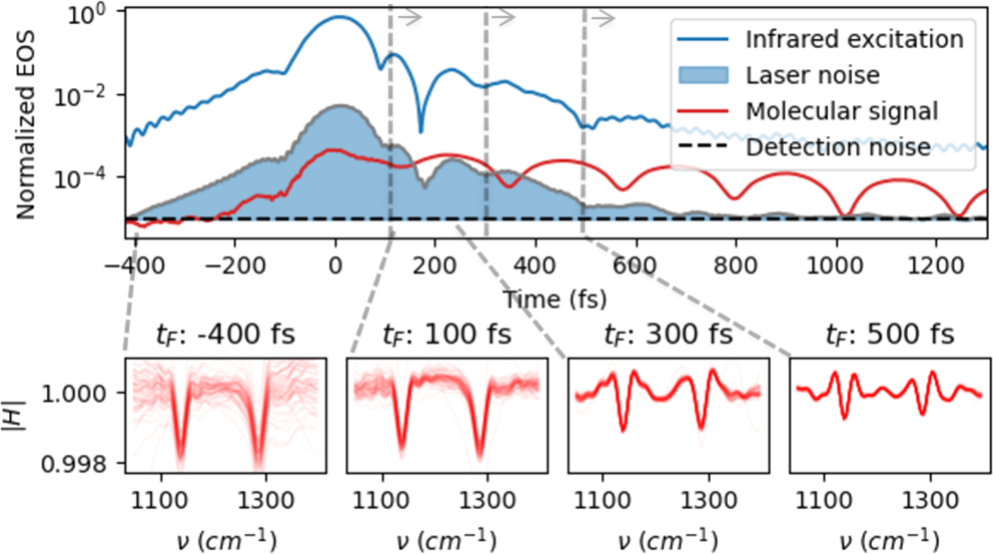

Field-resolved infrared spectroscopy (FRS) of impulsively excited molecular vibrations can surpass the sensitivity of conventional time-integrating spectroscopies, owing to a temporal separation of the molecular signal from the noisy excitation. However, the resonant response carrying the molecular signal of interest depends on both the amplitude and phase of the excitation, which can vary over time and across different instruments. To date, this has compromised the accuracy with which FRS measurements could be compared, which is a crucial factor for practical applications. Here, we utilize a data processing procedure that overcomes this shortcoming while preserving the sensitivity of FRS. We validate the approach for aqueous solutions of molecules. The employed approach is compatible with established processing and evaluation methods for the analysis of infrared spectra and can be applied to existing spectra from databases, facilitating the spread of FRS to new molecular analytical applications.

## Introduction

Vibrational spectroscopy has become an indispensable tool for a multitude of biological, chemical, and medical applications.^1−5^ Raman and infrared (IR) spectroscopy probes the molecular conformation and composition of a given organic specimen. The fact that virtually any solid, gas, or liquid sample can be investigated in a label-free manner with minimal sample preparation has promoted the widespread use of Raman and IR spectrometers.

An important factor contributing to the popularity of these techniques is that, when carefully measured and adequately processed,^6^ vibrational spectra are largely instrument-independent, and thus, quantitatively comparable. This has enabled databases containing hundreds of thousands of spectra of chemical substances in different physical states, for molecular identification and quantification of substances via chemometric approaches.^5,7,8^

Field-resolved infrared spectroscopy (FRS) of organic molecules^9^ has demonstrated favorable signal scaling for strongly absorbing samples^10^ as well as improved sensitivity compared to time-integrating infrared spectroscopy, such as Fourier transform infrared (FTIR). The distinctive feature of FRS is the ability to temporally record the electric field emanating from the molecules excited by few-cycle IR pulses^11^ with nearly single-photon sensitivity^12^ via electro-optic sampling (EOS).^13−15^ Thanks to the ultrabrief excitation, a large fraction of the molecular response is temporally separated from the much more intense excitation and thus also from its correspondingly strong multiplicative noise. This temporal confinement of noise is absent in conventional time-integrating spectroscopies and therefore not explicitly considered in standard data processing. This results in the processed signal being strongly contaminated by the technical noise of the excitation.

Previous studies^9,10^ have showcased the circumvention of this limitation for FRS by subtracting—directly in the time domain—a reference signal from the sample signal. This grants access to pure resonant signal emitted by the molecules in the sample and enables its separation from the noisy excitation, increasing sensitivity as compared to conventional data processing. Yet, the resulting “molecular fingerprint” depends on the spectral amplitude and phase distribution of the excitation pulse. This dependence on instrument parameters has so far limited the practical application of FRS in real-world scenarios.

Here, we employ an FRS data processing procedure that overcomes these limitations, delivering nominally excitation-pulse-independent broadband sample information. At the same time, the approach largely eliminates technical noise carried by excitation by exploiting its strong temporal confinement. This procedure is also applicable to FTIR data and compatible with all related spectral processing methods.^8,16^ This enables the use of established methods for the quantitative and qualitative analysis of FRS spectra as well as allowing direct comparison of FRS and FTIR data. The method presented here thus establishes important prerequisites for the practical application of FRS technology and can be regarded as a blueprint for standardized processing of FRS data.

## Experimental Section

### EOS Measurement and Noise Sources

**Figure 1:**
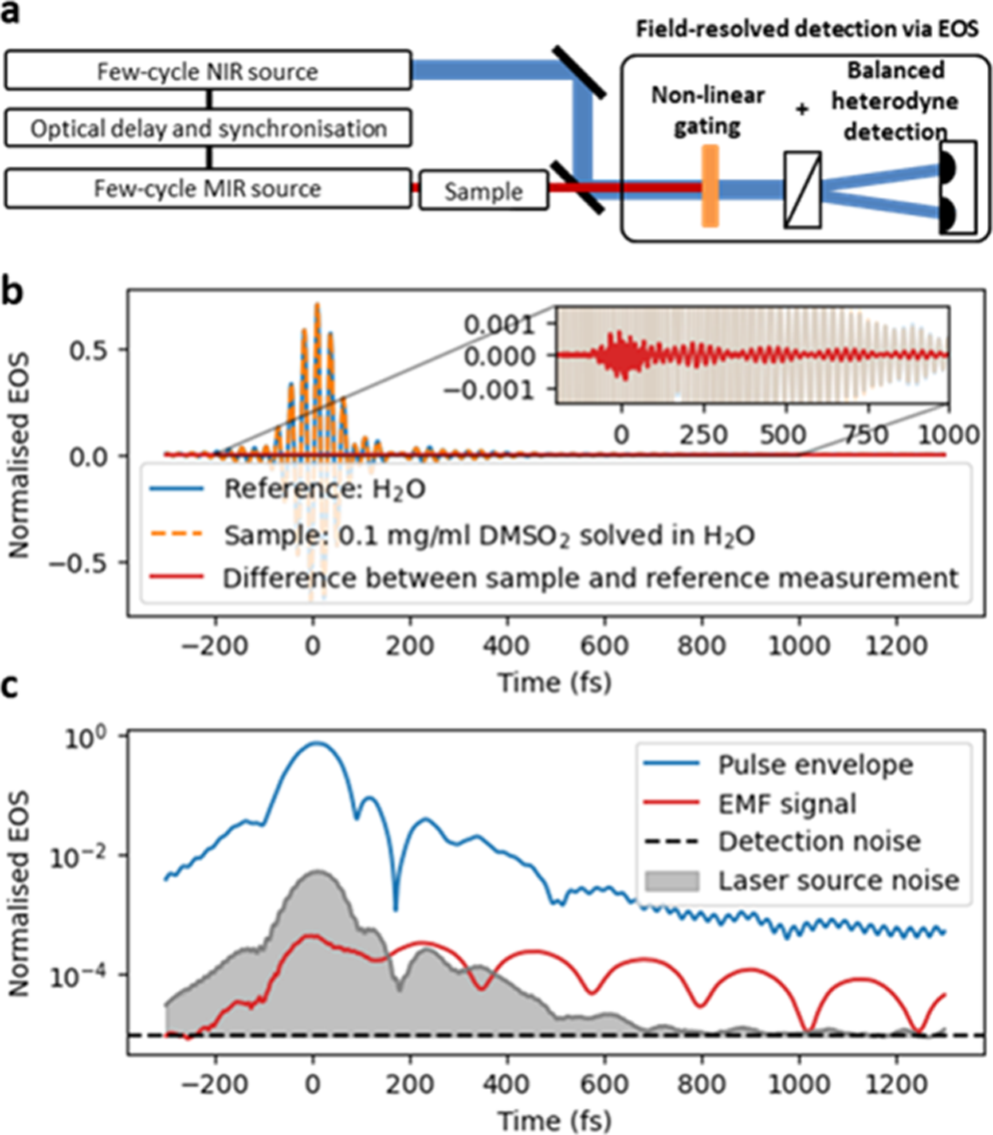
Field-resolved spectrometer and measurement characteristics. (a) Sketch of a typical field-resolving spectrometer. (b) Electro-optic sampling (EOS) measurements of a reference trace (H_2_O, blue) and a sample trace (DMSO_2_ solved in water, orange). The difference of the two signals represents the vibrational response of the investigated molecule(s) in the time domain (red). (c) Due to the field- and time-resolved nature of FRS, the molecular signal (red) and the detection noise (gray) have a characteristic temporal evolution that must be considered in order to extract molecular information with the highest possible signal-to-noise ratio (SNR). The excess noise follows the envelope of the exciting pulse (blue) and decays faster than the actual electric field molecular fingerprint (EMF) signal for the majority of the vibrational modes, resulting in a better SNR at the trailing edge of the pulse.

The schematic of an FRS instrument is shown in Figure 1a. The molecular sample, here, an aqueous solution contained in a cuvette, is excited by a train of waveform-stable few-cycle IR pulses. Variably delayed near-IR pulses gate the IR molecular response via EOS. The gate and sample excitation pulses may originate either from a single laser oscillator or from independent sources.^17−19^ In both cases, there are several options for technical implementation to control the delay between the two pulse trains. The two FRS instruments used in this work are described in more detail in the Methods section and the Supporting Information (SI).

Assuming the instrument response of EOS to be linear,^20^ the measured signal *S*_ref_(*t*) can be described as a convolution of the IR electric field *E*_0_(*t*) with an instrument response function IRF(*t*):^9^
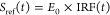
1

Note that in many cases the instrument response function may be rather flat, and therefore the EOS signal closely resembles the electric field (Figure 1b).^9,21,22^

When a sample is introduced into the beam path, the signal, under the assumption of linear excitation and in the absence of noise, can be modeled by the convolution with the linear function *H*(*t*):^9^
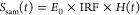
2

The function *H*(*t*) describes the instrument-independent (i.e., referenced) linear sample response in the time domain, thus, involving knowing or measuring a reference and a sample signal. For brevity, we henceforth refer to *H*(*t*) as the sample response. Note that *H*(*t*) only contains pure sample information when interference effects (e.g., between the walls of a thin cuvette) can be neglected. For a detailed description of how to extract the complex refractive index of materials from *H*(*t*), the interested reader is referred to other works.^23−27^ We would also like to point out that the sample response *H*(*t*) described here can be generalized for attenuated total resonance and reflection measurements.

In general, effects such as the interaction of substances with each other,^28,29^ the displaced water volume compared to the pure water reference measurement,^30,31^ the measurement geometry, and interference must be considered in order to obtain the pure material response of the substance dissolved in the water.^23−25^ However, in the case of low analyte concentrations, eqs 1 and 2 represent a sufficiently good approximation.^27^ The validity of this approximation over a wide concentration range implies that quantitative concentration retrieval of molecules dissolved in water is feasible via a linear fit over many orders of magnitude.^9^ Our treatment based on eqs 1 and 2 applies in the limit of low concentrations.

For a sufficiently short excitation pulse, a flat instrument response function and a weak sample response eq 2 becomes (as discussed in the Supporting Information Section II of our previous work^9^):
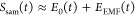
3Hereby, *E*_EMF_(*t*) denotes the sample-specific molecular electric field molecular fingerprint (EMF) of the sample, emitted in the wake of the excitation pulse.^9^ In many instances, this is a good approximation for FRS measurements, and the EMF can be obtained by subtracting the sample from the reference measurement in the time domain (Figure 1b). However, this EMF signal depends on the amplitude and phase of the excitation, which may vary over time for a given instrument and differ for different instruments. This impedes the comparison of EMF data recorded at different times or with different instruments.

The goal of this study was to develop a procedure allowing for the isolation of information from pure, excitation-pulse-independent sample response *H*(*t*) with the best possible signal-to-noise ratio (SNR). The achievable SNR is ultimately limited by the quantum efficiency of the detection system and by the optical shot noise.^32^ In practice, however, the noise level is typically dominated by other factors, such as detector noise or intensity fluctuations of the source. The performance of time-integrating spectroscopies (such as FTIR or direct absorption spectroscopy) is therefore often limited by the total (time-integrated) noise power of the source. In contrast, in FRS, only the fraction of the infrared radiation reaches the detector that is sampled by the nonlinear detection process.^9^ Consequently, the noise power within an EOS trace follows the envelope of the trace and is therefore temporally localized within the time window of the excitation (Figure 1c). An EOS trace affected by noise *S*_noise_(*t*) can be modeled using
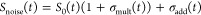
4where *S*_0_(*t*) is the theoretical noise-free signal (e.g., *S*_sam_(*t*) or *S*_ref_(*t*)), and σ_mult_(*t*) and σ_add_(*t*) are terms representing the contributions of multiplicative and additive noise, respectively. Typical contributions to multiplicative noise are relative intensity noise of the light source or beam-pointing fluctuations. Detector noise or shot noise can be modeled as additive noise.^32^ The model based on eq 4 reproduces the noise of typical measurements very well (Figure 1c). This analysis shows that after the excitation, the noise level drops to the detection noise (in the data displayed in Figure 1c about 700 fs after the excitation peak), creating ideal conditions for detecting the weak molecular signal with the highest possible sensitivity. In the following section, we discuss how this property of FRS can be used in data analysis in order to enhance the SNR of the measured molecular information, while reducing the dependence on the instrument settings.

### Standard Approach for Obtaining Molecular Spectra

Standard linear spectroscopy aims to isolate the pure, instrument-independent spectrally resolved sample response *H̃*(ω). Using the “standard approach”, this is performed in the frequency domain (FD), where a convolution in the time domain becomes a multiplication. The sample response *H̃*(ω) in the FD can be obtained by
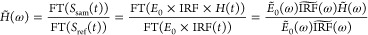
5

**Figure 2:**
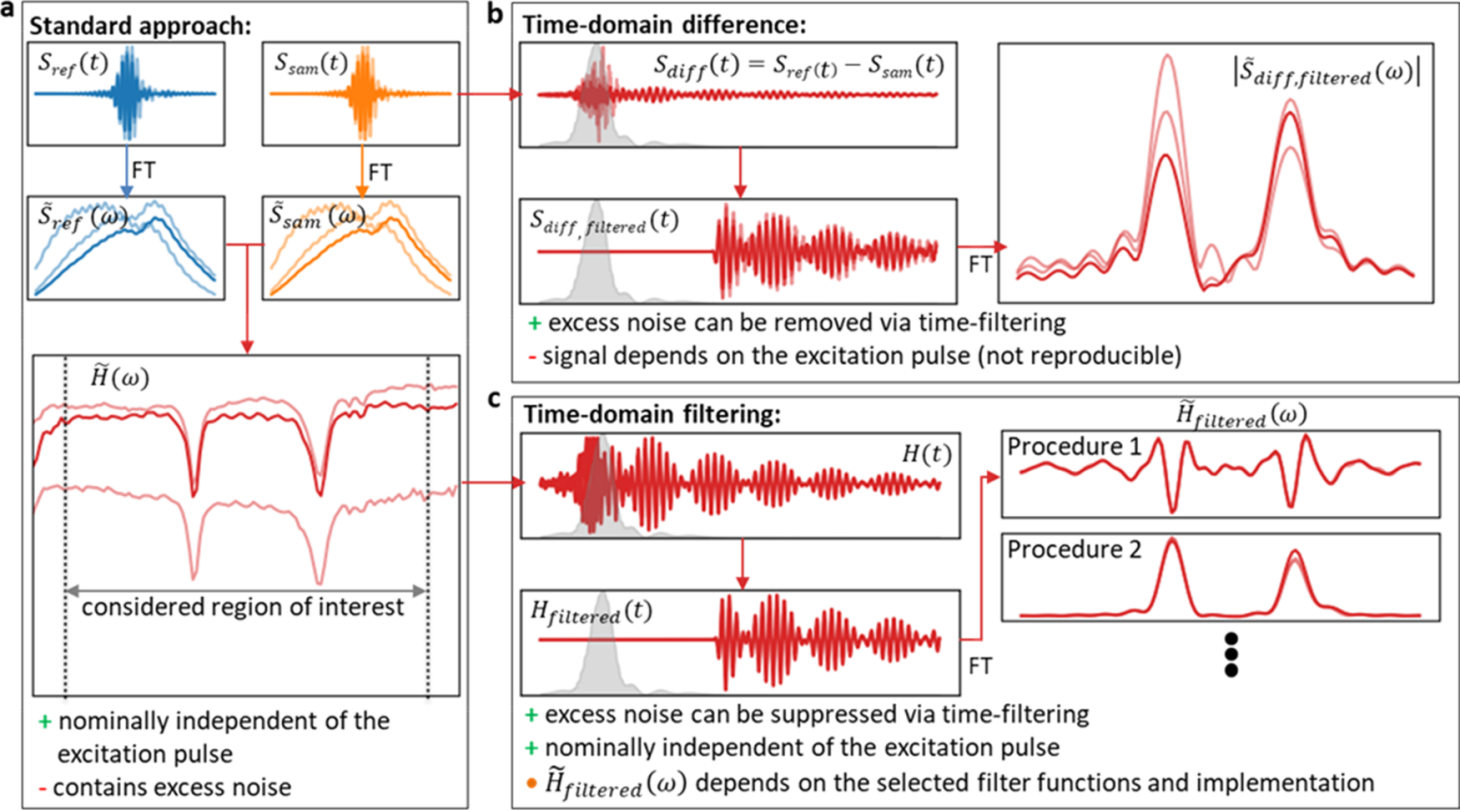
FRS data processing procedures for extracting molecular information. (a) The standard approach calculates the complex sample response *H̃*(ω) by dividing the sample and reference spectra (*S̃*_sam_(ω) and *S̃*_sam_(ω)) that are obtained via Fourier transformation of the measured EOS traces *S*_sam_(*t*) and *S*_ref_(*t*). While *H̃*(ω) is nominally independent of the excitation pulse *E*(*t*), considering the entire time trace transfers the excitation noise to the frequency domain, resulting in a degradation of the signal-to-noise ratio (SNR). (b) The excitation noise can be effectively suppressed by subtracting the original time traces *S*_sam_(*t*) and *S*_ref_(*t*) from each other and by applying a temporal filter at the location of the excitation (as indicated by the gray shaded area), resulting in superior molecular sensitivity.^9^ However, the filtered molecular response *S*_diff,filtered_(*t*) depends on the excitation pulse’s amplitude and phase, making it challenging to compare measurements with different instrument settings or devices. (c) The time-domain filtering (TDF) procedure follows the approach described in (b). However, temporal filtering is now applied to the time-domain representation *H*(*t*) of the sample response *H̃*(ω) (which is nominally independent of excitation *E*(*t*)), yielding reproducible results with superior SNR. (a–c) The transparent line indicates the outcomes of the different approaches for different excitation pulses *E*(*t*). The shaded gray areas in (b) and (c) indicate the time-dependent noise power.

Under the assumption that *Ẽ*_0_(ω) and  are identical for the reference and sample measurements (which is usually the case for sequential measurements close in time), *H̃*(ω) is nominally independent of the excitation. Although mathematically simple, the experimental determination of *H̃*(ω) may become inaccurate due to inevitable measurement noise and drifts (Figure 2a). Due to the fact that the entire time trace is considered, all technical noise contained is also transferred to the FD and, accordingly, the resulting sample spectra are heavily affected by it (as indicated by the transparent lines in Figure 2a). There are well-established data processing approaches in IR spectroscopy, able to remove measurement artifacts, baseline distortions or reduce the detrimental impact of noise on the spectra obtained,^8,16,33^ but, to the best of our knowledge, none of them adequately considers the characteristic temporal noise structure of an FRS measurement and allow it to be suppressed by a straightforward-comprehensible selection of filter parameters.

### Obtaining Molecular Information by Subtraction in the Time Domain

Considering the approximations made for the derivation of eq 3, the subtraction of the reference from the sample FRS measurements yields (“TD-differences”, Figure 2b):^9,10^
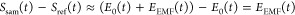
6

As described in eq 4, the dominant multiplicative noise present during the measurement follows the envelope of the excitation pulse *E*_0_(*t*) (Figure 1c, gray line) which is usually orders of magnitude stronger than the additive noise σ_add_(*t*), and limited to a time window of several 100 fs. At the same time, the sample-specific electric field *E*_EMF_(*t*) spans over several ps for most molecular vibrations (e.g., Figure 1c, red line). Consequently, when a time filter is applied to eq 6, the noise introduced by the excitation can be drastically reduced while preserving the majority of the molecular signal *E*_EMF_(*t*) (Figure 2b). In our previous work, this time filter was set to the value at which the multiplicative noise reaches the detection noise floor (e.g., at approximately 700 fs for the example displayed in Figure 1), which was key to achieving unprecedented sensitivity in the optical detection of molecular vibrations.^9,10^

Although this approach can effectively suppress technical noise and increase the measurement SNR as compared to the standard approach, the instrument-independent sample response *H*(*t*) is not recovered because *E*_EMF_(*t*) depends on *E*_0_(*t*), which, in turn, depends on the instrument and is subject to temporal variations (usually on a slow time scale). This limits the practical applicability, as discussed below in more detail.

### Time-Domain Fourier Filtering of Molecular Spectra

The time-domain filtering (TDF) approach (Figure 2c) is applied to the sample response *H̃*(ω) obtained with the standard approach (eq 5). It uses the fact that the temporal structure of the noise (eq 4 and Figure 1c) is transferred to the FD in a characteristic way. The temporal localization of the noise leads to “low-frequency” oscillations in the reference and sample spectra, whereby the width of these oscillatory features corresponds to the inverse temporal width of the localized noise in the TD. These features of the individual measurements are also transferred to the obtained referenced sample response *H̃*(ω). Although there exist approaches to reduce slow modulations of the baseline of *H̃*(ω),^32,33^ these methods do not allow to precisely account for a given, particular temporal structure of the excitation noise.

Here, we utilize a procedure that overcomes this shortcoming while simultaneously taking advantage of the strong temporal localization of the noise. A detailed (mathematical) description and discussion of the processing pipeline, as well as considerations for its practical application, and the effects of the exact implementation of TDF can be found in the Supporting Information and the accompanying code. In the following, we outline the main steps and underlying ideas. First, the temporal representation of the sample response, *H*(*t*), is calculated via an inverse Fourier transformation:
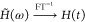
7

This transformation naturally projects the noise in the TD into the region it originated from to the temporal region of the excitation, around time zero. We can now apply a time filter *w*(*t*) to *H*(*t*)—see Figure 2c—in a similar way as we did before to *S*_diff_(*t*) in Figure 2b. The result is then transformed back to the FD to yield the time-filtered sample transfer function *H̃*_filtered_(ω), sketched in Figure 2c,right. The full procedure reads:
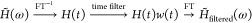
8

**Figure 3:**
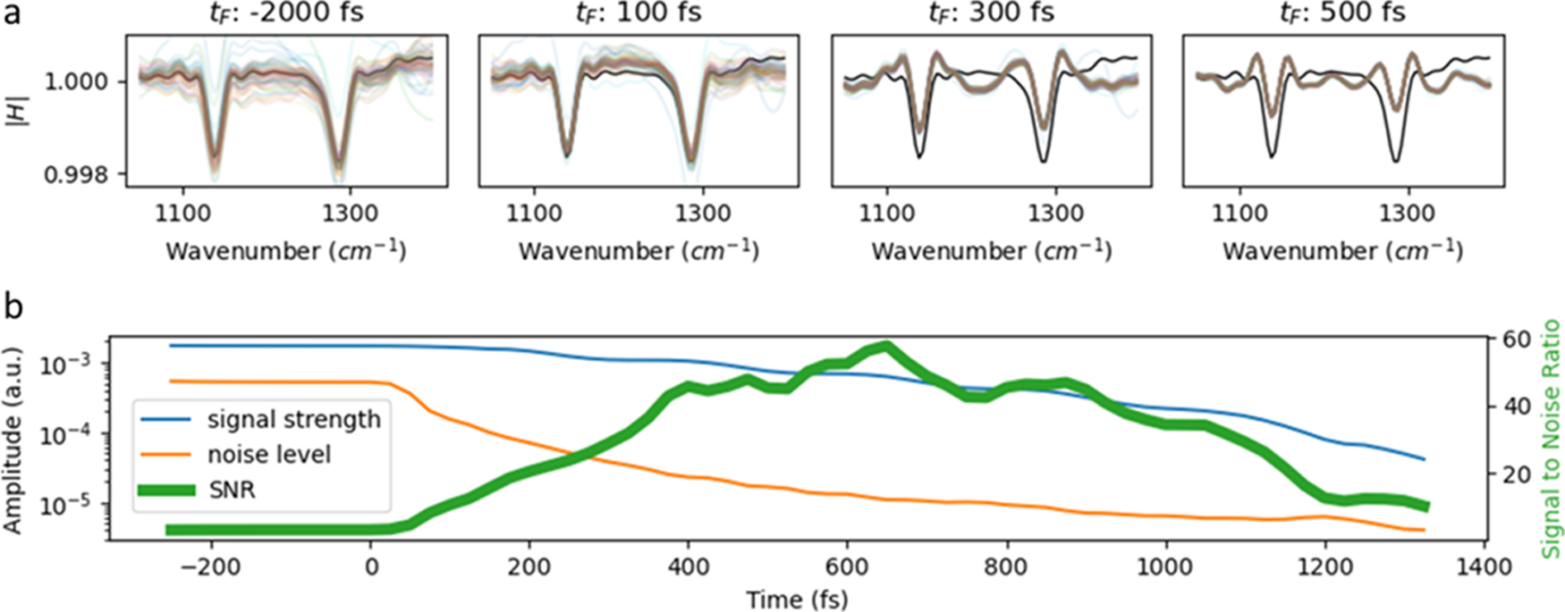
Effect of time-domain filtering on the spectrum, signal strength, noise level, and signal-to-noise ratio (SNR). (a) 59 measurements of 0.1 mg/mL DMSO_2_ solved in water were taken over a time span of over 2 years for different types of time-domain filter. With the increasing value of the cutoff of the high-pass Heaviside filter, the signal strength as well as the noise decrease while the spectrum is altered more strongly. The mean of the unfiltered spectra is depicted as a black line. (b) Evolution of the signal strength (maximum deviation from 1 of the filtered DMSO_2_ spectra as depicted in (a), noise level (standard deviation (frequency-averaged between 1000 and 1400 cm^–1^) of filtered spectra of measurements pure water), and signal strength to noise ratio (SNR) in dependence of the chosen cutoff).

It is important to note that the above expression describes only the underlying idea of TDF. It can be implemented in different ways with different filter functions, which may have advantages and disadvantages depending on the application (see the SI for a brief discussion). Thereby, TDF may strongly alter the original signal according to the chosen type of implementation (Figures 2c and 3a). The crucial point, however, is that with a fixed implementation of TDF, this procedure can deliver sample-specific, and nominally excitation-pulse-independent molecular fingerprints with reduced measurement noise contributions from the excitation pulse and, thus, with improved SNR. Furthermore, TDF is a linear method. This means that if the original spectra can be described by a linear combination of different individual spectra, the time-filtered spectrum is a linear combination of time-filtered individual spectra. This also implies that time-filtered spectra are suitable for linear regression methods for the determination of molecular concentrations.

The underlying concept of TDF is mathematically similar to the well-known technique of Fourier self-deconvolution that is widely applied in FTIR processing for band-narrowing^8,16,34,35^ or the application of Fourier transform methods for baseline correction.^33^ The main difference in TDF is the type of filter applied in the time domain. In Fourier self-deconvolution, the filters are designed to enhance the separation of peaks of overlapping bands, while in the above-mentioned Fourier transform method, the filters are used to correct for distortions of the baseline. In contrast, the filters in the TDF approach are specifically designed to suppress the multiplicative noise of the excitation source.

We have chosen the simplest possible implementation of the TDF for all of the results presented in this paper. First, a frequency filter with smooth transitions is applied to the sample transfer function to select the spectrum within the region of interest. Next, the TD representation is calculated. A Heaviside filter is then applied because the hard cut permits us to clearly assess how much of the original noise still contributes to the filtered signal. Finally, the result is transferred back to the FD under consideration of the original offset component of the sample transfer function.

Figure 3 illustrates how the shape and SNR of a typical spectrum are altered when a Heaviside filter function with increasing cutoff is chosen. The highest SNR is achieved at a cutoff of 650 fs, which approximately corresponds to the value at which the multiplicative noise reaches the measurement noise floor (Figure 1c), in accordance with our earlier studies.^9,10^ Thus, a noise analysis of the FRS data can guide the choice of cutoff values for the time filter. The fringes next to the absorption-band frequencies are an effect of the chosen implementation. By applying more sophisticated filters, such as a Butterworth filter with appropriate parameters, these oscillations can be further suppressed. Alternatively, the magnitude of the filtered spectrum can also be analyzed (Figure 2c—Procedure 2). Although the information about the sign of the absorption is lost, the observed fringes are effectively suppressed (Supporting Figure S4). This can be advantageous for spectral interpretation or band fitting. Further examples of different TDF implementations and their effects on the filtered spectra and the SNR can be found in the Supporting Information.

## Results

In the following, we experimentally investigate the performance of the TDF approach in comparison to those of the other methods discussed. We show that TDF can also be applied to conventional IR spectra and demonstrate that spectra from databases containing absorption spectra can be utilized for the evaluation and interpretation of TDF-FRS data. It should be noted that spectral data recorded with FRS are inherently complex, comprising the spectral amplitude and phase information. For simplicity, and to allow for a direct comparison to FTIR data, we only analyze the magnitude of the complex FRS data in the remainder of this work.

### Concentration Retrieval

**Figure 4:**
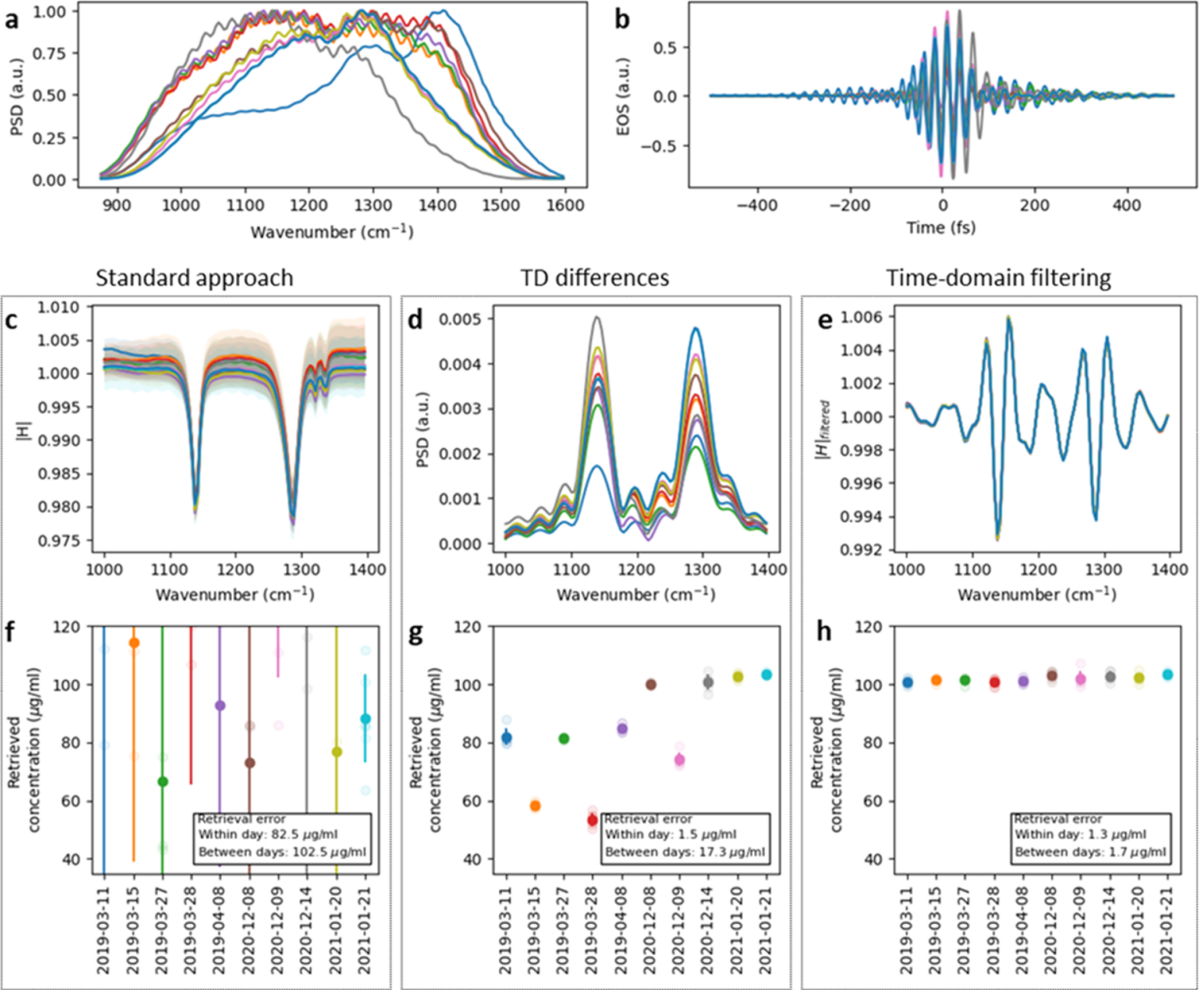
Performance of the three different data processing approaches with different instrument settings. A DMSO_2_ dilution series was measured on different days over a period of 2 years with different configurations of the FRS instrument. (a, b) Spectra and time-domain signal of the corresponding excitation pulses. (c–e) The obtained frequency response of 1000 μg/mL DMSO_2_ was solved in water for the three different data processing approaches. The colored lines and the shaded areas represent the mean value and the error (standard deviation) of a measurement series, respectively. (f–h) Concentration retrieval of 100 μg/mL DMSO_2_ was conducted in water. The 1000 μg/mL spectra of measurement day 10 were used as a reference spectrum within the concentration retrieval procedure (see the Methodssection). (a–e) Each color represents the results for one measurement day.

For benchmarking purposes, we apply TDF together with the two other methods (standard approach and TD-differences) to the analysis of 10 data sets of FRS signals of DMSO_2_ solved in water that were repeatedly measured over a period of 2 years (see the Methods). Within this time frame, several adaptations were made to the FRS instrument (replacement of nonlinear crystals, optics, detectors, etc.), which introduced substantial changes in instrument parameters. Therefore, measurements taken at different times can be considered to have been taken by different devices. This is also reflected by the substantial variation of the temporal and spectral shapes of the respective excitation pulses (Figure 4a,b).

The molecular spectra obtained are displayed in Figure 4c–e. The “TD difference” method can strongly suppress the technical noise (recognizable by the small standard deviation within a measurement day), but the retrieved molecular signals obtained on different days vary significantly. In contrast, the standard approach yields comparable spectra but with a large retrieval error. The best results, in terms of precision (standard deviation within a measurement day) and reproducibility of the spectra, are achieved with the TDF approach.

Similar conclusions can be drawn when retrieving the concentration from measurements of a 100 μg/mL solution of DMSO_2_ in water, when using the spectrum of 1000 μg/mL DMSO_2_ measured once at a specific single experimental day as reference (Figure 4f–h). While the results obtained with the standard approach and TD difference approach exhibit either a large retrieval error or a strong variation between measurement days, respectively, the TDF data processing approach yields excellent results across the entire extended measurement campaign.

Note that the retrieval error of the standard approach can be significantly improved by performing a baseline correction before the concentration retrieval (see the Supporting Information for the results obtained with various baseline correction methods). While baseline correction methods have the advantages of largely preserving the band shape and facilitating spectral interpretation, these methods are usually not designed to specifically account for the characteristic time-domain structure of noise, such as in FRS measurements. Moreover, the choice of filter parameters of such methods in order to best suppress the characteristic noise in FRS measurements is not straightforward. Therefore, the results obtained from the TDF approach are still significantly better than the results obtained with baseline correction methods. The results presented in this work have a slightly larger instrument error compared to our previous work,^9^ as an instrument regime favoring long-term stability over sensitivity was chosen for the measurements presented.

### Compatibility with Existing Processing Pipelines and Use of Infrared Database Spectra

In the previous section, we have shown that time-domain filtering of FRS data significantly increases the SNR of individual measurements, as well as their reproducibility over different measurement days. Although the filter may change the shape of the spectrum considerably, for many applications this is unproblematic, because a corresponding reference spectrum (e.g., recorded with a different device) can be subjected to the same filtering for comparison. As a result, spectra originating from infrared databases can also be used to interpret TD-filtered FRS data.

**Figure 5:**
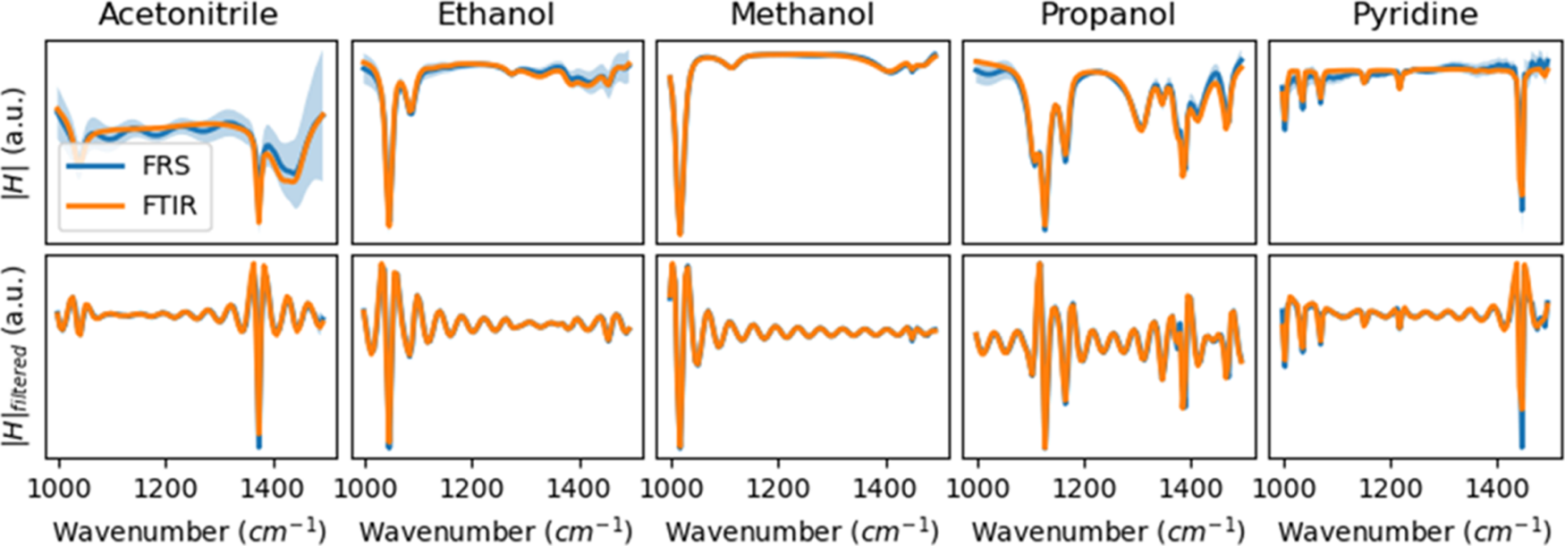
Compatibility of time-domain filtered FRS and conventional infrared absorption data. Top row: FRS and FTIR measurements of five different common solvents mixed with water were done in a volume ratio of 1:99%. Bottom row: The same spectra were obtained after applying a high-pass Heaviside Fourier filter at 700 fs. Each substance was measured five times with each instrument. The colored area indicates the measurement error. The spectra are offset corrected and normalized for better comparability.

To showcase this, we measured five different common solvents mixed with water in a volume ratio of 1:99% with a commercial FTIR spectrometer (Microbiolytics GmbH) and an FRS instrument and compared the obtained spectra (Figure 5 and the Methods section for details). The unprocessed FRS and FTIR spectra already show good agreement; however, some systematic deviations are discernible. These are likely due to different interference effects caused by the use of sample cuvettes with different layer thicknesses (7 vs 33 μm) and different window materials (CaF_2_ vs ZnSe) for the FTIR and FRS measurements, respectively. Such a typical interference pattern can be most prominently seen in the 1100–1300 cm^–1^ range in the FRS measurement of acetonitrile.

Without time-filtering FRS data, i.e., with the standard approach, the measurement uncertainty is significantly higher than in the corresponding FTIR spectra. When applying TDF, the relative error of the FRS measurements decreases (blue shaded areas in Figure 5). In addition, the comparability of the measurements taken with both devices drastically improves. Most of the systematic differences between FRS and FTIR measurements, which are caused by interference or baseline shifts, are strongly reduced.

The cause of the reduction of the interference effect can be understood in the time domain. The first echo of the excitation pulse caused by the cuvette windows lies approximately 280 fs behind the excitation. By applying a high-pass filter at 700 fs, this echo is filtered out, strongly reducing the influence of multiple reflections on the TDF spectrum. This example shows that TDF not only increases the SNR of FRS measurements but can also reduce interference effects, altogether rendering FRS and FTIR measurement more comparable.

Despite the observed improvement, the influence of interference effects on the filtered spectra cannot be completely eliminated due to differences in the geometry and type of liquid cuvette used. Such spectral deviations can become problematic in many real-world biochemical, biological, and biomedical applications, which often rely on machine learning computational models^6,36^ that are prone to picking up systematic patterns. This problem, which is well known in IR and Raman spectroscopy, can be partially resolved through proper preprocessing and model building.^6,36,37^ However, cross-laboratory and cross-device comparisons remain a challenge,^38,39^ and further advancements in preanalytical sample preparation, measurement cuvettes (e.g., Brewster-angled windows), and data processing^40^ may be necessary to fully overcome this issue.

**Figure 6:**
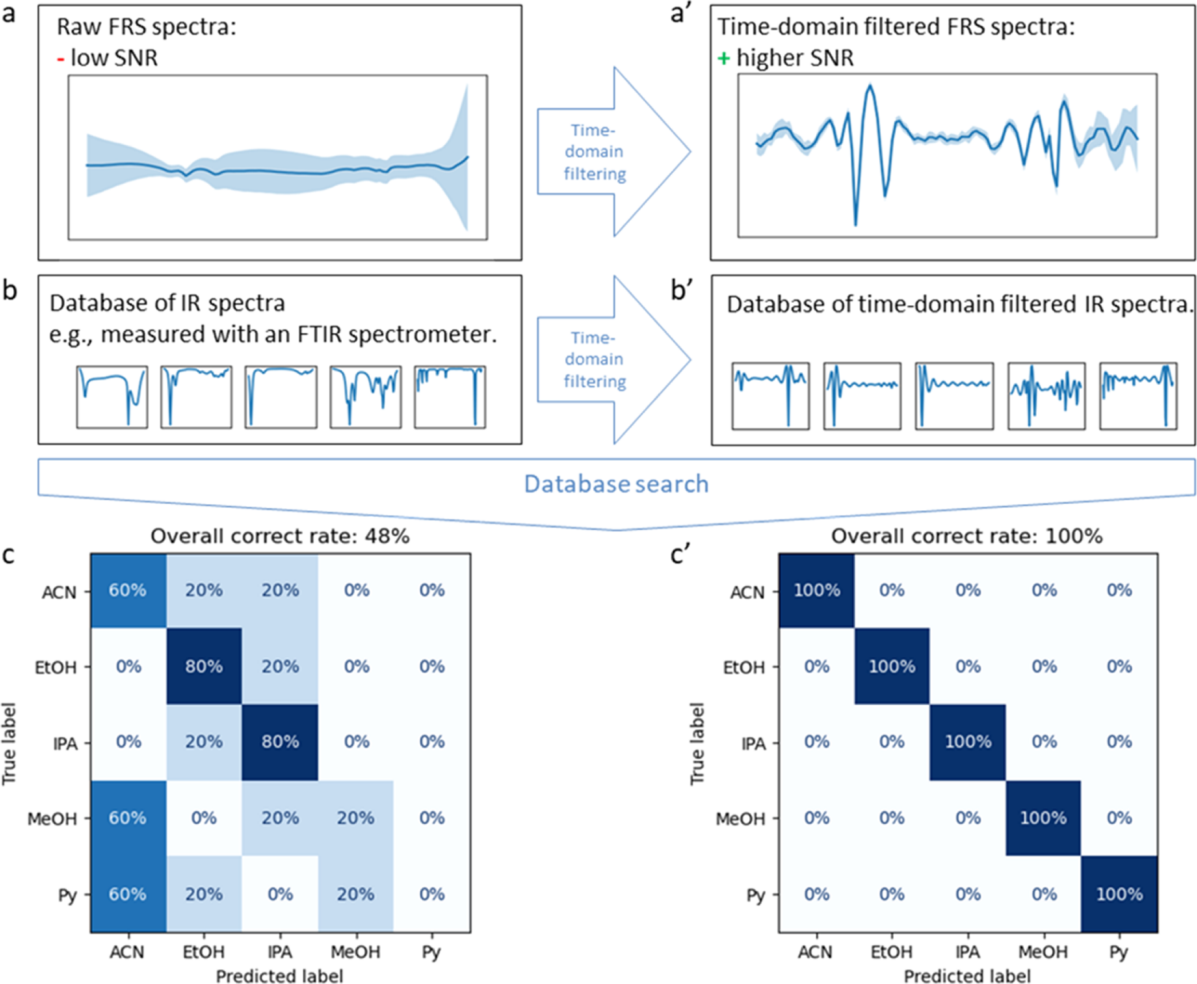
Case example: Application of infrared databases for trace analyte identification with FRS. Five solvents were mixed with water in a low mixing ratio, and each solution was measured five times using an FRS instrument. The aim was to evaluate how well the samples could be identified based on their measured FRS spectra. (a) Due to the low signal-to-noise ratio, a comparison with (b) a database of infrared spectra for (c) the identification of the substances was not unambiguously possible. (a′–c′) After applying a time filter to the measured spectra and the infrared database, the correct rate of identification increased to 100%.

Next, we show that this compatibility can be used to implement molecular identification based on FRS spectra that are compared with a database of FTIR spectra. To this end, we measured the five solvent–water mixtures with FRS at a concentration of 0.01 vol %, which poses a challenge for ordinary FTIR spectrometers (Figure 6a). The reference database was therefore assembled from FTIR measurements at a 100 times higher concentration of 1 vol % (Figure 6b). Each of the measured spectra was matched against the database using a simple search algorithm based on cosine similarity (see the Methods section for details), and the most likely substance was determined. In the case of working with the unprocessed referenced sample response, the overall correct rate is only 48% due to the non-negligible noise of the excitation pulse (Figure 6c).

Applying TDF to the FRS spectra in order to remove most of the excitation noise, thereby increasing the SNR of spectra, yielded the results plotted in Figure 6a′. The same TDF was applied to the FTIR database (Figure 6b′), and afterward, the database search was performed again (Figure 6c′). Due to the increased SNR of the filtered spectra, a 100% correct rate was achieved.

## Conclusions

Infrared FRS is a powerful novel spectroscopic method that enables precise measurements of molecular signals at the level of the electric field. To exploit the full potential of FRS, preprocessing procedures are required that account for the characteristic noise structure of the time-resolved FRS measurements.

In this work, we demonstrated that noise carried by the ultrashort excitation pulse can be effectively removed by applying a TDF to the measured referenced sample response, thereby substantially increasing the SNR of the measured molecular signal. The highest SNR is achieved approximately at the time when the multiplicative noise of the excitation pulse reaches the detection noise. This makes it possible to find favorable parameters for the application of the TDF by means of a simple noise analysis. In contrast to previous data processing approaches for FRS data, time-filtering is not applied to the difference in the original time traces, which depends on the shape of the excitation pulse. Instead, it is applied to the TD representation of the genuine, nominally excitation-pulse-independent sample response. This enables the comparison of FRS fingerprints of spectra taken with different pulse parameters or even different types of infrared spectrometers.

We further demonstrated that TDF can also be applied to FTIR data and that the resulting spectra can be utilized for the evaluation and interpretation of TDF-FRS spectra. This compatibility with established data processing routines widens the scope of the proposed approach and enables direct comparison of FRS data with, e.g., spectra from IR databases.

The presented data processing procedure is expected to be essential in promoting the broad applicability of the FRS technology. Along with foreseeable technological advances, it is likely to impact further applications of FRS in life sciences, biochemistry, and molecular medicine.

## Methods

### Experimental Setup

The experimental results used within this work were taken with two different field-resolved spectroscopy (FRS) setups. For clarity, we name them infrared (ISA) 1 and 2. A detailed description of ISA 1 can be found in our previous work.^9,10^ ISA 2 can be regarded as a further development of ISR 1 and shares many of its technological concepts. Both spectrometers are driven by Yb:YAG thin disk oscillators providing ultrashort pulses at a central wavelength of 1 μm with 100-W-scale average powers. These pulses are further shortened to sub-20 fs durations by spectral broadening via self-phase modulation in multiple passes through bulk media and subsequent compression with dispersive mirrors. In both devices, broadband mid-infrared radiation is obtained by intrapulse-difference-frequency generation in LiGaS_2_ crystals. The electro-optic detection setups in ISA 1 and 2 were both equipped with GaSe crystals and balanced detection. The main difference between ISA 1 and 2 is the delay scanning mechanism employed, including the type of gate pulses used for electro-optical sampling (EOS): While ISA 1 uses a mechanical delay stage and a copy of the temporally compressed 1 μm pulses also used for mid-infrared generation, ISA 2 uses the pulses of an erbium-doped fiber laser together with electro-optic delay tracking for the acquisition of EOS traces at a rate of 2.8 kHz.^17^

### Recording Spectra of Liquids

The FRS measurements of liquids were performed with both instruments using an automated liquid sample handling and delivery system (microbioloytics GmbH, customized design). For the actual spectroscopic measurement, the liquid is injected into a cuvette (microbioloytics GmbH) consisting of two ZnSe windows with a path length of approximately 34 μm, optimized for maximum sensitivity.^10^ Throughout the measurement campaign, different cuvettes with slightly different path lengths were used. This was considered in the evaluation of the spectra by calibrating the actual path length for each measurement day by measuring a liquid with known absorption (1 mg/mL DMSO_2_ solved in water) and scaling the measured spectra accordingly. For each liquid sample, a reference spectrum (water in the cuvette) and a sample spectrum (sample in the cuvette) were recorded. The effective measurement time for the sample and the reference was 40 s each. The measured time window is 8.3 and 5.8 ps for ISR 1 and 2, corresponding to a spectral resolution of 4 and 5.7 cm^–1^, respectively.

### Data Processing and Time-Domain Filtering

The exact data processing and detailed implementation of the time-domain filter (TDF) can be found in the provided code used for the analysis of the data. The Supporting Information contains additional considerations that may be helpful in the application of the TDF.

### DMSO_2_ Measurements and Concentration Retrieval

Ten sets of measurements of aqueous dimethyl sulfone (DMSO_2_) were performed with ISR 1 over a period of 2 years. Each set contained between 2 and 6 samples with a concentration of 1 mg/mL and between 5 and 15 samples with a concentration of 0.1 mg/mL. For concentration retrieval, the average of the 1 mg/mL measurements from the last sample set was used as reference. The concentrations of the measured samples were then determined by calculating a scalar product of the reference and the measured fingerprint of the sample. The exact procedure can be obtained from the data set and evaluation code provided. The retrieval errors specified in Figure 4 are the mean value of all standard deviations of the concentration values within a measurement day and the standard deviation of all retrieved concentration values across days.

### Database Search

The database of five infrared spectra used here was assembled using a commercial FTIR spectrometer (microbiolytics GmbH). Five replicates of acetonitrile, ethanol, isopropanol, methanol, and pyridine, each mixed with water with a volume ratio of 1:99% were each measured for 45 s and then averaged to obtain a reference spectrum of the respective substance. The FRS measurements of the solvents were performed with ISR 2. For the spectral sample identification, we used a simple search algorithm based on cosine similarity. In a first step, the FRS and database spectra were interpolated to the same frequency grid, and the same preprocessing steps were performed in each case. The mean value was then subtracted from each spectrum (centering). Next, the cosine similarity was calculated between a given measurement and each database spectrum. The database substance with the highest cosine similarity was assigned to the sample measured via FRS. The detailed procedure is provided in the evaluation code.

## Data Availability

Data set and Python Scripts: https://github.com/marinusHub/time-domain-filtering.

## Supporting Information Available

Additional considerations when applying time-domain filtering and additional comparisons of time-domain filtering to other preprocessing procedures (PDF)
