# Supplementary material for: Standardized Electric-Field-Resolved Molecular Fingerprinting
Source: Anal Chem. 2024 Jul 29;96(32):13110–9. doi: 10.1021/acs.analchem.4c01745 (PMC11325294; doi:10.1021/acs.analchem.4c01745)
Supplement: Supplementary file 1 — ac4c01745_si_001.pdf [file ac4c01745_si_001.pdf]

Supplementary Information

Standardized electric-field-resolved molecular fingerprinting

Authors:

Marinus Huber[1,2,3,4,5], M. Trubetskov[1,2], W. Schweinberger[1,2,6], P. Jacob[1,2], M. Zigman[1,2,6], F. Krausz[1,2,6], I. Pupeza[1,2,3,4,5,7,*]

Affiliations:

1 Max Planck Institute of Quantum Optics, 85748 Garching, Germany;

2 Ludwig Maximilian University of Munich, Department of Physics, 85748 Garching, Germany;

3 Leibniz Institute of Photonic Technology - Member of the research alliance "Leibniz Health Technologies", 07745 Jena, Germany

4 Cluster of Excellence Balance of the Microverse, Friedrich Schiller University Jena, 07743 Jena, Germany

5 Physics Department and State Research Center OPTIMAS, University of Kaiserslautern-Landau, 67663 Kaiserslautern, Germany

6 Center for Molecular Fingerprinting, 1093 Budapest, Hungary

7 Fraunhofer Institute for Industrial Mathematics ITWM, 67663 Kaiserslautern, Germany

* ioachim.pupeza@rptu.de.

Considerations when applying time-domain filtering

The following section discusses important considerations for the application of the concept of time-domain filtering (TDF), as well as various implementations of TDF using examples. This is not a complete overview, but is intended to give the interested reader an idea for how the TDF concept should be applied and what needs to be considered. The following points are discussed:

1. How to deal with non-zero baselines
2. Appropriate choice of the spectral region of interest
3. Complex- versus real-valued spectra
4. Effect of the shape of the time-filter
5. Analyzing the magnitude of complex time-domain filtered spectra

1. How to deal with non-zero-baselines

The proposed filter is essentially a high-pass filter. This means that any DC-component will be removed and therefore will change the DC level of the filtered spectra dramatically (see Figure S1a-d). This is the case, for example, when working with (complex) transmission spectra that are close to 1 for small absorption values. If the baseline is to be preserved, one can evaluate the DC level before applying the TDF and then add it back to the filtered result (Figure S2e-h). For small absorptions, a DC value of 1 can also be assumed and subsequently added after filtering. This feature is used in the analysis of the data provided in this paper.

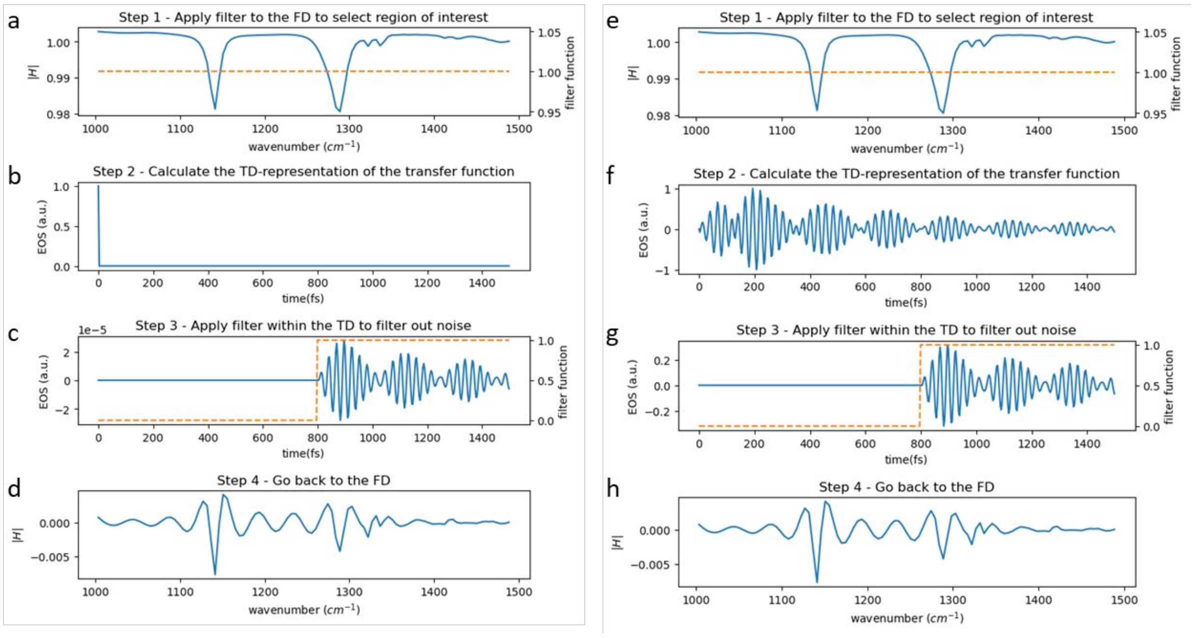

Fig. S1. Effect of time-domain filtering on baselines. *The time-domain filter inherently removes any DC baselines. For example, if the input signal or spectra is centered around 1 (a), a delta-pulse-like signal will appear in the corresponding time-domain representation at 0 fs (b). This peak will be removed during time-filtering (c), and consequently, the filtered signal will be centered around 0 (d). The original baseline can be preserved by calculating the baseline of the input spectra, and adding it back to the filtered spectra (e-h).*

2. Appropriate choice of the spectral range

A measured spectrum may not be well defined in the entire calculated spectral range due to limited spectral coverage of the light source, strong sample absorption, or other effects. This often leads to strong noise in these regions, which is often noticeable as a fast, random change between small and large values of the spectrum. If these noisy regions are not excluded before applying the TDF, they can affect and deteriorate the results in the actual region of interest (ROI) due to the non-local nature of TDF (Figure S2a-d). Therefore, it is recommended to set the value outside the ROI to the DC-value of the ROI (Figure S2e-h) or only keep the ROI as input to the TDF.

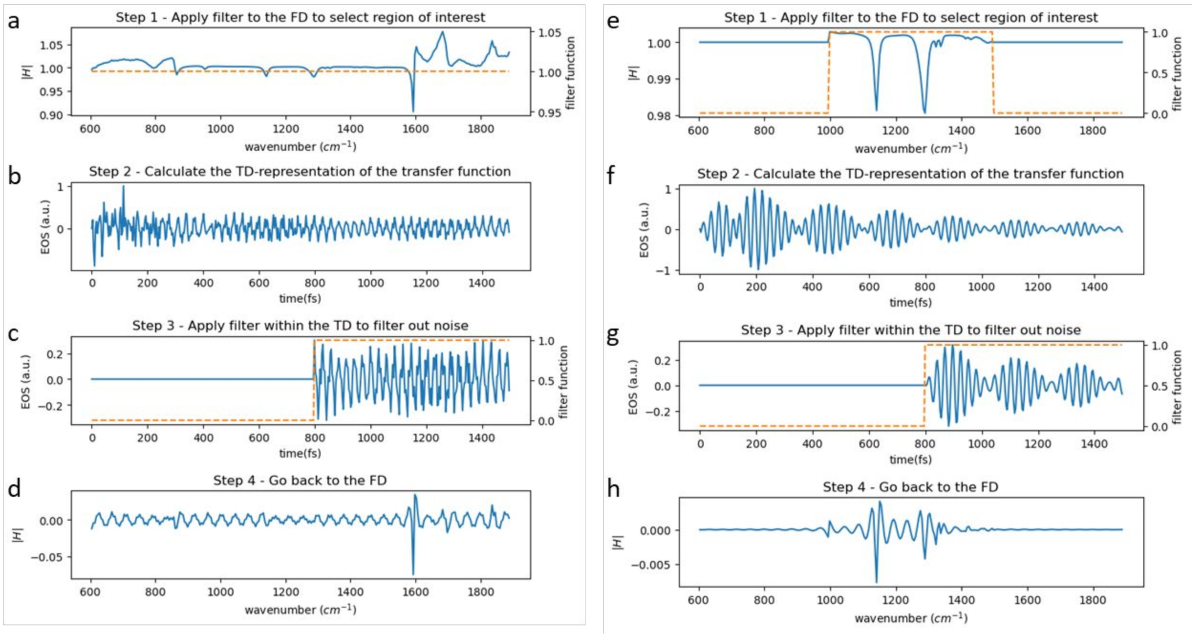

Fig. S2. Effect of the selected spectral range before applying the time-domain filter. *a-d: The considered spectral range also includes wavenumber in which the spectrum could not be calculated correctly due to the limited spectral coverage of the utilized light source. This may lead to artefacts in the filtered spectrum (d). e-h: This can be avoided by applying a spectral filter before applying the actual time-domain filter.*

3. Complex-valued versus real-valued spectra

Spectra obtained with FRS are naturally complex-valued and the concept of TDF can be directly applied to complex spectra. However, in this work, we chose to implement the TDF in a such a way, that real and imaginary parts of the spectrum are treated independently. This implementation offers more flexibility, since different types of processing (e.g. baseline correction, different values and types of the high-pass filter) can be chosen for the real and imaginary part separately, if necessary.

4. Effect of the shape of the time filter

For the sake of simplicity and to showcase the general applicability of the approach, only a Heaviside time-domain filter was used for the results presented in the main part of this work. However, other types of commonly used filters can be applied. . For example, a 1st-order Butterworth filter can significantly decrease the fringes next to absorption lines, since it dampens the roll-off in the filtered spectra (see Figure S3b). On the other hand, this also means that in the TD the cut-off is not sharp, and more noise from the excitation (which is centered around 0 fs) might be picked up.

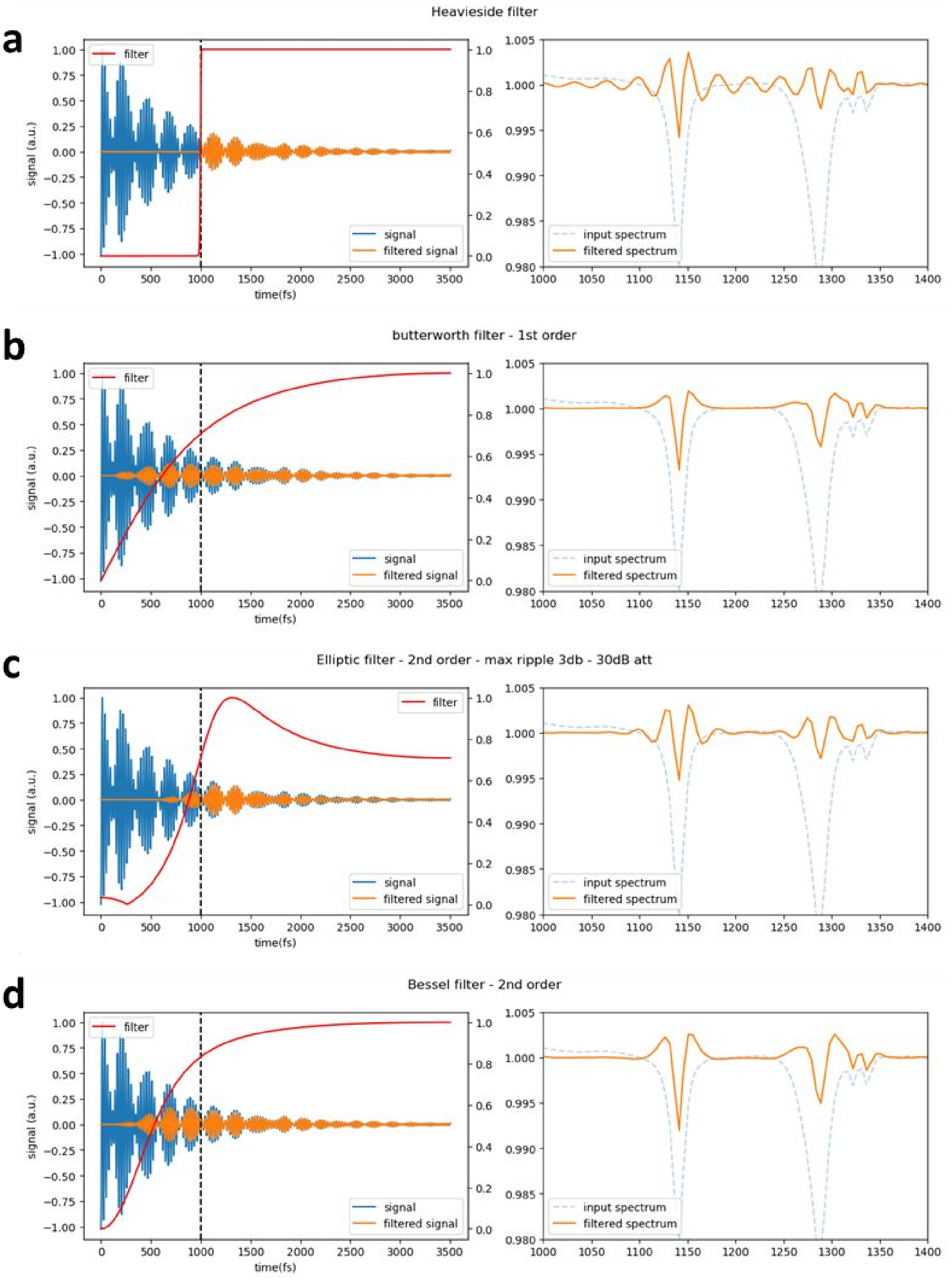

Fig. S3. Time-domain filtering with various commonly used filter functions.

5. Analyzing the magnitude of complex time-domain filtered spectra

Under point 3, we discussed that it can be advantageous to consider the real and imaginary parts of the spectra separately as real input spectra. Alternatively, the filter can be applied to complex input spectra. This produces a complex-valued time-filtered spectrum from which the power spectrum can be analyzed. Although one loses the information about the sign of the absorption, the oscillations next to the main bands are strongly suppressed (Figure S4a). In addition, the spectra filtered in this way have a greater similarity to the typical absorption spectra, which makes spectral interpretation easier.

In addition, similar to our previous work[1], a fit of the analyzed band can be performed to determine the dephasing time (Extended Data Figure 6 and 7 of reference 1).

The performance of this implementation for the experiments discussed in the manuscript is similar to the implementation used in the main part (Figure S4 b and c).)

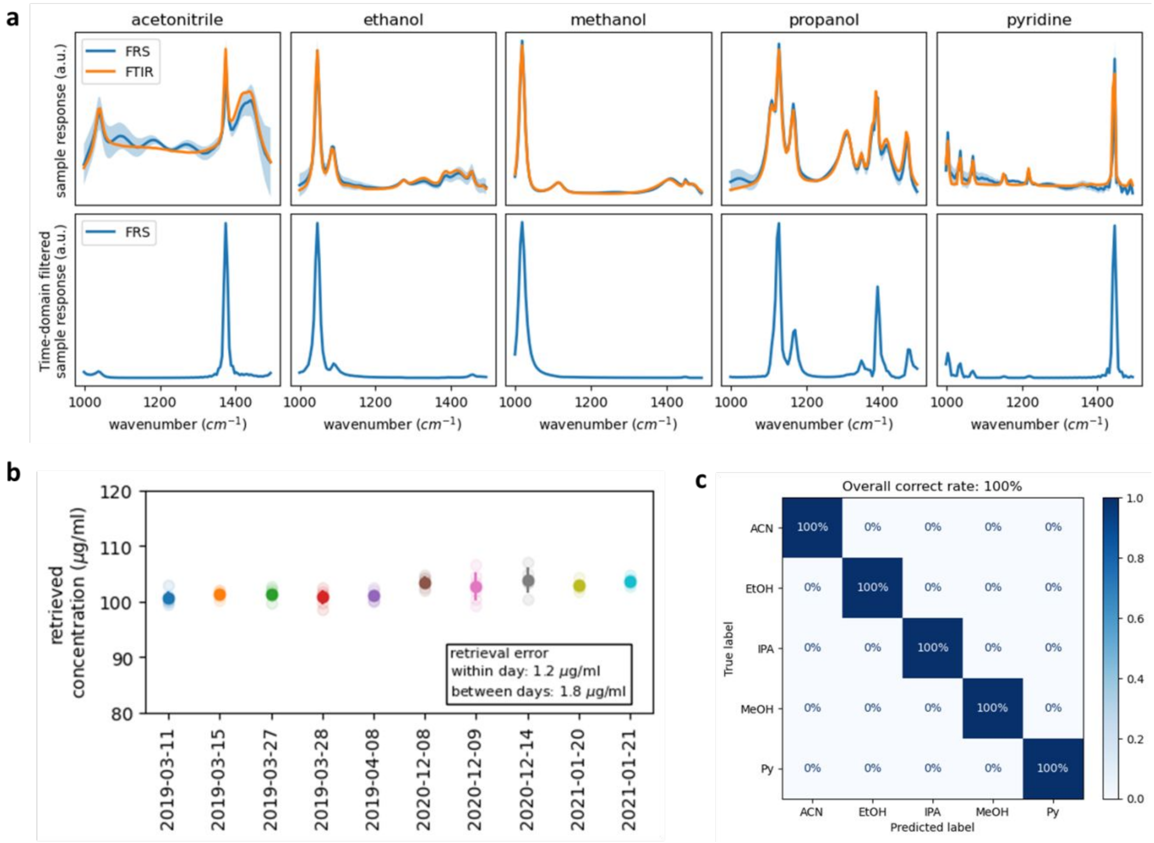

Fig. S4. Analyzing the magnitude of complex time-domain filtered spectra. a: *The top row shows the same results as displayed in Figure 5 of the main paper. The bottom row shows the magnitude of the time-filtered complex input spectra.* ***b,c****: Performance of the concentration retrieval and chemical identification experiment when using the magnitude of the time-filtered complex input spectra.*

Comparison of the approaches with additional pre-processing

It is widely recognized that infrared spectra can be prone to measurement inaccuracies or artefacts, but these can be mitigated through appropriate data processing. Several established data processing methods are already available. To enable a fair comparison between the standard approach and the time-domain (Fourier-)filtering (TDF) method, we applied various baseline correction methods to the spectra obtained using the standard approach. In comparison to the analysis presented in Figure 4 of the main paper, this analysis was performed on absorbance data so that available baseline correction methods could be readily applied. We tested several methods that are provided within the python module pybaselines[2] (see Table S1). The best performance was achieved with the improved modified polynomial baseline algorithm[5]. This correction reduced the error in quantifying $DSMO_2$ from around 80 to 3.7 ug/mL. Still, TDF provided the best performance.

Table S1: Comparison of the accuracy of the concentration retrieval using different pre-processing approaches.	
Pre-processing	Concentration retrieval
**Time-domain-filtering**	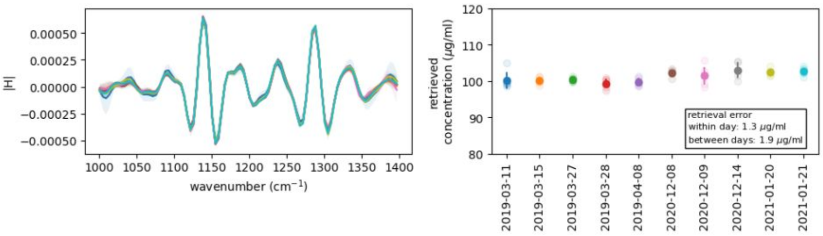
**Linear baseline correction**	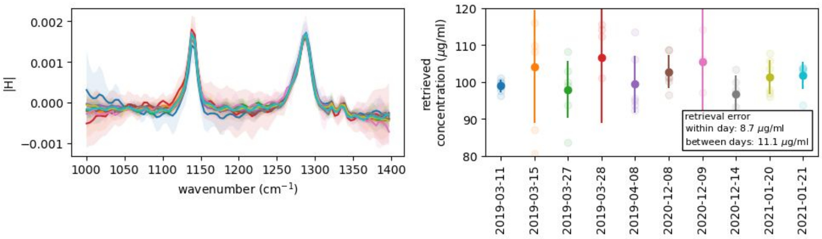
**Mixture Model**[3]	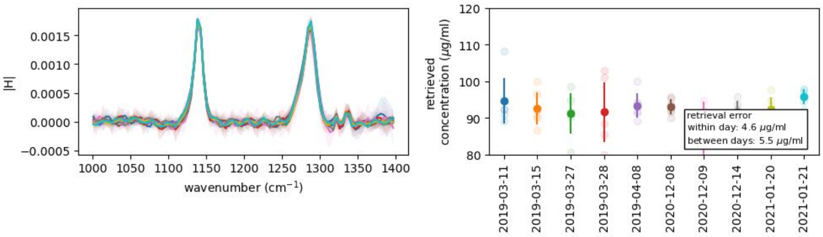
**Iterative Reweighted Spline Quantile Regression**[4]	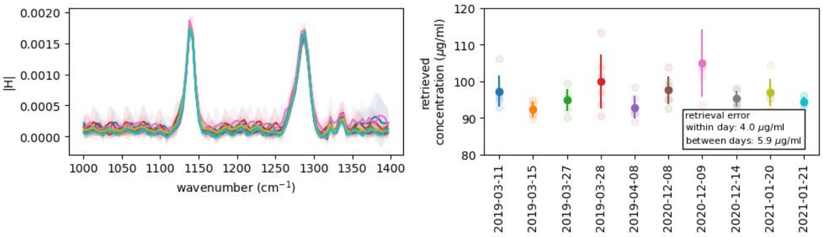
**The improved modofied polynomial (IModPoly) baseline algorithm**[5]	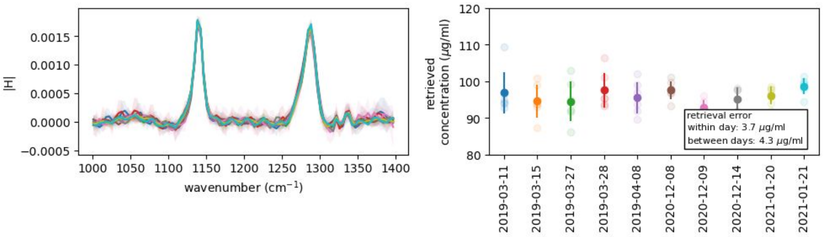
**A penalized spline version of the asPLS algorithm**[6]	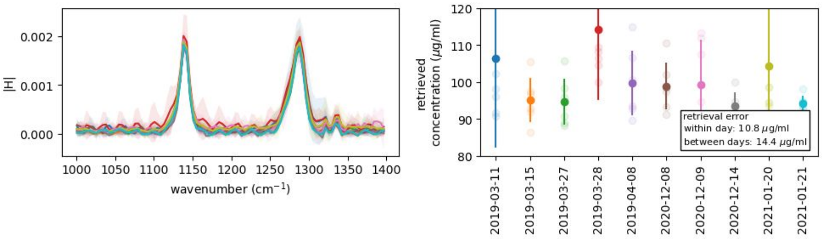
**Adaptive smoothness penalized least squares smoothing (asPLS)** 6	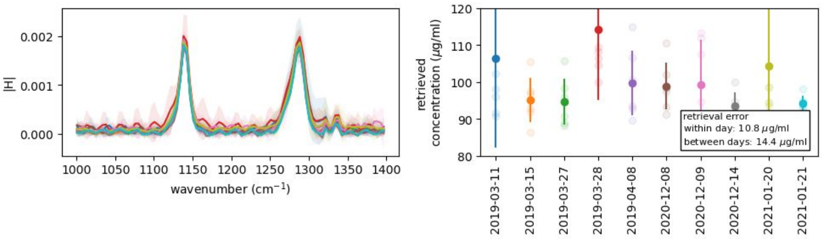

References:

1. Pupeza, I. *et al.* Field-resolved infrared spectroscopy of biological systems. *Nature* **577**, 52–59 (2020).

2. Erb, D. pybaselines: A Python library of algorithms for the baseline correction of experimental data. doi:10.5281/zenodo.5608581

3. de Rooi, J. J. & Eilers, P. H. C. Mixture models for baseline estimation. *Chemom. Intell. Lab. Syst.* **117**, 56–60 (2012).

4. Han, Q., Peng, S., Xie, Q., Wu, Y. & Zhang, G. Iterative Reweighted Quantile Regression Using Augmented Lagrangian Optimization for Baseline Correction. *Proc. - 2018 5th Int. Conf. Inf. Sci. Control Eng. ICISCE 2018* 280–284 (2018). doi:10.1109/ICISCE.2018.00066

5. Gan, F., Ruan, G. & Mo, J. Baseline correction by improved iterative polynomial fitting with automatic threshold. *Chemom. Intell. Lab. Syst.* **82**, 59–65 (2006).

6. Zhang, F. *et al.* Baseline correction for infrared spectra using adaptive smoothness parameter penalized least squares method. *Spectrosc. Lett.* **53**, 222–233 (2020).
